# Evaluation of Isomotive Insulator-Based Dielectrophoretic Device by Measuring the Particle Velocity

**DOI:** 10.3390/s22041533

**Published:** 2022-02-16

**Authors:** Ryu Nakabayashi, Masanori Eguchi

**Affiliations:** 1 Advanced Course, Project Design Engineering, National Institute of Technology (KOSEN), Kure College, 2-2-11, Aga-Minami, Kure, Hiroshima 737-8506, Japan; s20-ruby@kure.kosen-ac.jp; 2 Department of Electrical Engineering and Information Science, National Institute of Technology (KOSEN), Kure College, 2-2-11, Aga-Minami, Kure, Hiroshima 737-8506, Japan

**Keywords:** single-cell analysis, isomotive dielectrophoresis, insulator-based dielectrophoresis, particle tracking velocimetry, particle characterization

## Abstract

Many dielectrophoretic (DEP) devices for biomedical application have been suggested, such as the separation, concentration, and detection of biological cells or molecules. Most of these devices utilize the difference in their DEP properties. However, single-cell analysis is required to evaluate individual properties. Therefore, this paper proposed a modified isomotive insulator-based DEP (iDEP) creek-gap device for straightforward single-cell analysis, which is capable of measurement at a wide frequency band. The proposed iDEP device generates more constant particle velocity than the previous study. The insulator was fabricated using backside exposure for accurate forming. We measured the distribution of the particle velocity and frequency property, using homogeneous polystyrene particles to verify the effectiveness of the proposed device. The results show that the particle velocity distribution was consistent with the distribution of the numerically calculated electric field square ($\nabla E_{\mathrm{rms}}^{2}$). Furthermore, the velocity measurement, at a wide frequency band, from 10 Hz to 20 MHz, was performed because of the long distance between electrodes. These results suggest that the prop-erties of various particles or cells can be obtained by simple measurement using the proposed device.

## 1. Introduction

Dielectrophoresis (DEP) is an electrokinetic phenomenon, which is the movement of a polarizable microparticle suspended in a medium under a non-uniform alternating current (AC) electric field [1]. The movement of particles toward a high electric field is called positive DEP (pDEP), whereas their movement away from this field is called negative DEP (nDEP). The DEP force acting on the particles depends on the radius of the particles, complex permittivity of the particle and medium, and gradient of electric field square ($\nabla E_{\mathrm{rms}}^{2}$).

The properties of the particles driven by DEP, such as biological cells, are characterized by the DEP collection rate [2], equilibrium point between DEP force and gravity [3,4], cross-over frequency [5], and terminal velocity of particles [6]. Generally, particles have unique DEP properties with frequency dependence, due to their structural and electrical properties. Therefore, these properties can be evaluated through the DEP properties. Moreover, there are various biomedical applications of DEP utilizing the difference in the DEP properties of particles, such as the separation of human cancer cells from normal cells [7] and live and dead cells [8], the concentration of human cancer cells [9], and detection of deoxyribonucleic acid labeled to microbeads [10]. Therefore, the measurement of the DEP properties of particles or cells is one of the subjects for DEP application.

Cellular heterogeneity in isogenic cell populations has been widely observed, e.g., in stem cells [11]. Thus, it is crucial for cell analysis to measure at a single-cell level, representing individual property, instead of averaged properties, through bulk measurement [12]. In the electrical perspective, the frequency properties of cells contain much information, such as structural and electrical properties [13]. The literature stated that the electrical properties of cell membranes might be prognostic markers for tumor detection and treatments because they are remarkably changed by malignant transformation, affecting their growth [14].

Recently, several methods have been developed to characterize particles or cells using DEP [15,16], traveling wave DEP (twDEP) [15], and electrorotation (ROT) [17,18,19]. These devices are electrode-based (eDEP) systems. Therefore, the measurement at low frequency is limited, due to the electric double layer (EDL) at the interface between electrode and medium. Moreover, cells may be damaged by a high electric field [20], such as near the electrode. However, the 3DEP platform is a rapid and high-accuracy measurement method for the DEP properties of cells [21]. Further, it is unsuitable for single-cell analysis, since it achieves high-accuracy measurements by averaging the results derived from multiple samples.

More recently, isomotive DEP (isoDEP) devices were developed as the straightforward single-cell analysis method. isoDEP devices have two modes: eDEP and insulator-based DEP (iDEP). On iDEP devices, an electric field is generated by electrodes, and a non-uniform electric field is formed by insulators. Therefore, iDEP devices can easily be prevented from the damage of cells [22] because the test region of iDEP device is away from the electrodes. The pDEP-exhibited particles adhere to the edge of the electrode in eDEP device, due to the concentration of the electric field to electrodes. Meanwhile, it is less of a problem in iDEP [23]. In isoDEP, particles move on the constant $\nabla E_{\mathrm{rms}}^{2}$, leading to a constant particle velocity. This concept was first suggested by Pohl [24]. It was later demonstrated with polar and rectangular coordinate systems by Allen et al. [25] and Tada et al. [23,26], respectively. The particles moving on isomotive field are characterized by their velocity, and their electrical properties are enabled to evaluate by the velocity. The device with a polar coordinate system has a chamber with a 120° bend angle, and the particles move toward radius. In contrast, the device with a rectangular coordinate system has a creek-gap-shaped insulator, and the particles move on the centerline between the insulators. 

**Figure 1:**
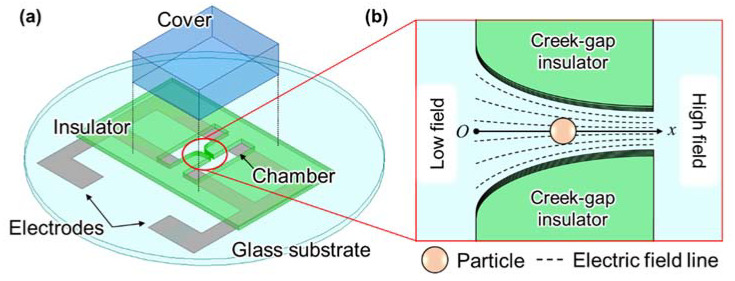
Illustrations of the proposed isomotive iDEP creek-gap device. (**a**) The isometric view of the entire device. (**b**) The enlarged top view of the chamber. A non-uniform electric field was formed by changing the gap between creek-gap insulators.

In this study, we proposed a modified isomotive iDEP creek-gap device. Figure 1 shows the concept of the proposed device. The electrodes generate an electric field, and a non-uniform electric field is formed by changing the gap between creek-gap insulators at the center of the chamber. This concept of the proposed device is the same as in our previous study [23]. However, the design guideline of the device was modified to realize a more constant $\nabla E_{\mathrm{rms}}^{2}$. Furthermore, the validity of numerically calculated $\nabla E_{\mathrm{rms}}^{2}$ was confirmed through the consistency of the distribution between the measured particle velocity and $\nabla E_{\mathrm{rms}}^{2}$. After that, the particle velocity measurement was performed at a wide frequency band from 10 Hz to 20 MHz. Velocity decay was observed at a very low frequency (≤100 Hz). Additionally, the cause was explained using the equivalent circuit of the proposed device, including the EDL between the electrode and medium.

## 2. Theory and Device Designing

### 2.1. Particle Velocity Induced by the Dielectrophoretic Force

The time-averaged dielectrophoretic force, $\langle F_{\mathrm{DEP}}\rangle$, is calculated according to [27], as follows:

(1) 〈FDEP〉=2πε0εmR3Re[CM*]∇Erms2

where $\varepsilon_{0}$ and $\varepsilon_{m}$ are the free-space permittivity and relative permittivity of suspending medium, respectively; *R* is the particle radius; $\nabla E_{\mathrm{rms}}^{2}$ is the gradient of the electric field square; Re[CM*] is the real part of Clausius–Mossotti factor, ${\mathrm{CM}}^{*}$, which is expressed by:

(2) $${\mathrm{CM}}^{*}=\frac{\varepsilon_{p}^{*}-{\;\varepsilon }_{m}^{*}}{\varepsilon_{p}^{*}{+2\varepsilon }_{m}^{*}}$$

where $\varepsilon_{p}^{*}\;\mathrm{and}\;\varepsilon_{m}^{*}$ are the complex permittivity of the particle and suspending medium, respectively. The complex permittivity, $\varepsilon^{*}$, is given by:

(3) $$\varepsilon^{*}=\varepsilon \;-\;j\frac{\sigma }{\omega }\;$$

where ε and σ are the permittivity and conductivity, respectively; *j*^2^ = −1; ω is the angular frequency. 

The drag force, *F_d_*, acting on spherical particles is given by Stokes’ law, as follows:

(4) $$F_{d}=6\pi \eta \mathrm{Rv}$$

where η is the viscosity of the medium, and v is the relative velocity of the particle.

Additionally, the friction force, *F_f_*, acting on the particle surface is given by:

(5) $$F_{f}=\frac{4}{3}{\pi R}^{3}{(\rho }_{p}-{\;\rho }_{m})\mu g$$

where $\rho_{p}$ and $\rho_{m}$ are the mass density of the particle and suspending medium, respectively; μ is the coefficient of the dynamic friction, and g is the gravitational acceleration.

Suppose $F_{\mathrm{DEP}}$ and $F_{f}$ are constant. The governing equation for the particle moving on a plane is expressed as a function of time, *t*, as follows:

(6) $$\;\frac{4}{3}{\pi \rho }_{p}R^{3}\frac{dv(t)}{dt}{=\langle F}_{\mathrm{DEP}}\rangle -{\;F}_{d}(v(t))\;-{\;F}_{f}$$

When the initial velocity of the particle is zero and *y*-component of DEP force is balanced on the *x*-axis, due to its symmetry, the particle velocity is described as follows:

(7)  v(t)=R23η{ε0εmRe[CM*]∂Erms2∂x−23(ρp − ρm)μg}{1 −exp(−tτ)}

where τ is the time constant in the particle motion on the device, which is given by:

(8) $$\tau =\frac{{2\rho }_{p}R^{2}}{9\eta }\;$$

If the frictional force is negligible because it is smaller than the x-component of the DEP force and t≫τ, then v is given by:

(9)  v(t)=ε0εmR2Re[CM*]3η∂Erms2∂x

Under the above assumption, the particle velocity is proportional to $\partial E_{\mathrm{rms}}^{2}$/∂x (i.e., on the constant $\partial E_{\mathrm{rms}}^{2}$/∂x, the particle behavior is the isomotive movement).

### 2.2. Design of the Proposal Isomotive iDEP Device

#### 2.2.1. Analysis of the Device Having Parallel Insulators

**Figure 2:**
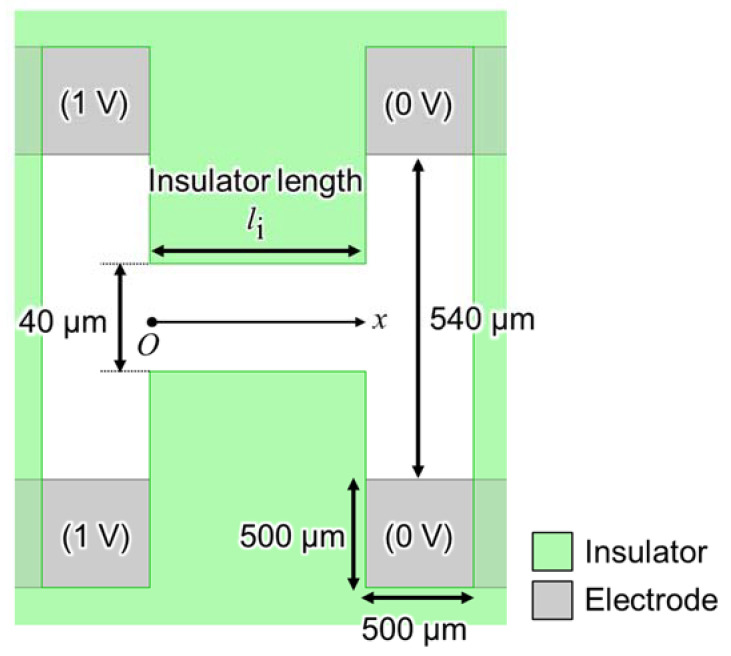
Top view of the device with parallel insulators for analyzing the generated electric field properties.

eDEP and iDEP devices form an isomotive field by changing the gap between the electrodes and insulators, respectively. There are clear theories for the electrode design of eDEP devices, such as E=V/d (where *V* is the applied voltage and *d* is the distance between electrodes at a parallel-plate electrode), but not for the insulator of iDEP devices. The device with parallel insulators (Figure 2) was numerically calculated to analyze the properties of the generated electric field between insulators using electromagnetic field simulation software (Ansys Maxwell, ANSYS, USA). It has the same construction as the device shown in Figure 1; however, only the shape of insulators was changed from creek-gap to parallel insulator. The device’s materials are the substrate (glass with 0.5 mm-thickness), electrodes (aluminum), insulator (SU-8 with 70 µm-thickness), and cover (silicone rubber with 100 µm-thickness). More detailed material information is presented in Section 3. For the device’s parameters, the gap of the insulators is 40 µm; the distance between the same potential electrode is 540 µm; the length of each side of the electrode uncovered with the insulator is 500 µm; the insulator length is defined as the variable. The voltage applied to the electrodes was setup as shown in Figure 2. Herein, the calculated fields were read at the height of 10 µm from the substrate because the radius of the used particle was 10 µm.

**Figure 3:**
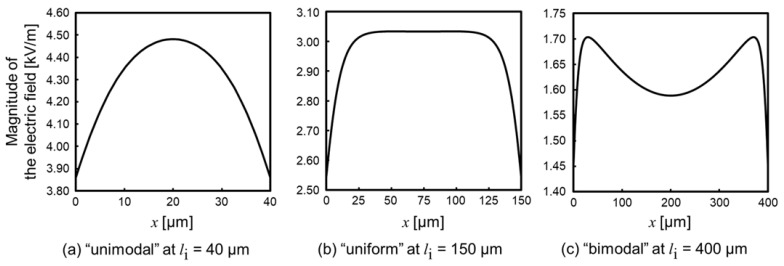
Generated electric field properties between the centerline and parallel insulators of the device. (**a**) The “unimodal” electric field at *l*_i_ = 40 µm. The distribution has one maximal point of the electric field. (**b**) The “uniform” electric field at *l*_i_ = 150 µm. The distribution partly has a uniform electric field. (**c**) The “bimodal” electric field at *l*_i_ = 400 µm. The distribution has two maximal points of the electric field.

The ideal property of the generated electric field on the centerline between insulators is “uniform” because the intensity of the electric field line does not change, according to the concept of the iDEP device. However, three patterns of the properties (Figure 3) were observed and called “unimodal”, which has one point of maximal electric field (Figure 3a), i.e., “uniform”, which partly has a uniform electric field (Figure 3b) and “bimodal”, which has two points of maximal electric field (Figure 3c) by changing *l*_i_. The uniform distribution was observed when 90 µm ≤ *l*_i_ ≤ 210 µm. If *l*_i_ is shorter than 90 µm, the property is unimodal, due to the lack of insulator length for gathering the electric field line. Meanwhile, if *l*_i_ is longer than 210 µm, the property is bimodal, due to the concentration of the electric field around the sidewall of the insulator, at near *x* = *l*_i_/2. Furthermore, the properties of the generated electric field did not almost change when the device was scaled up. Thus, the properties were seemingly determined by the ratio of the device’s parameter, e.g., the gap of insulators and insulator length.

#### 2.2.2. Design of Isomotive iDEP Creek-Gap Device

Following the above analyses, the “uniform” electric field is generated on the device, shown in Figure 2, when 90 µm ≤ *l*_i_ ≤ 210 µm. Furthermore, the properties were determined by the ratio of the device’s parameter. Therefore, the isomotive iDEP creek-gap device was re-designed by matching the ratio of each parameter to the device, with a parallel insulator using the gap between insulators as the reference. First, the shape of creek-gap insulators was determined. The range of 90 µm ≤ *l_i_* ≤ 210 µm is also described as 2.25 ≤ *l*_i_/*g* ≤ 5.25, using a gap of insulators, *g* = 40 µm. If the insulator length is 250 µm, 2.25 ≤ *l*_i_/*g* ≤ 5.25 is denoted as 48 µm ≤ *g* ≤ 111 µm (i.e., 2.25 = *l*_i_/*g*, then *g* = 250 µm/2.25 ≈ 48 µm, due to the insulator length of 250 µm). The shape of creek-gap insulators followed the equation of the design guideline of isomotive eDEP creek-gap device and was given as [26]:

(10) $$y=\frac{V_{\mathrm{rms}}}{2\sqrt{\mathrm{ax}+b}}\;$$

where $V_{\mathrm{rms}}$ is the root mean square of the applied voltage; *a* is $\partial E_{\mathrm{rms}}^{2}$/∂x, and *b* is the constant determined by the boundary conditions in the isomotive eDEP device. Equation (10) is also used for the isomotive iDEP creek-gap device.

Next, we determined the distance between the same potential electrode, *d*_e_, and length of each side of the electrode uncovered with the insulator, *l*_e_. According to the device with parallel insulators, and since *d*_e_ = 540 µm and *l*_e_ = 500 µm, we obtained *d*_e_/*g* = 13.5 and *l*_e_/*g* = 12.5. Thus, the shape of the electrode is determined as *d*_e_≈ 650 µm and *l*_e_ ≈ 600 µm on the part of the narrower *g*, as well as *d*_e_ ≈ 1500 µm and *l*_e_ ≈ 1390 µm on the part of the wider *g*.

**Figure 4:**
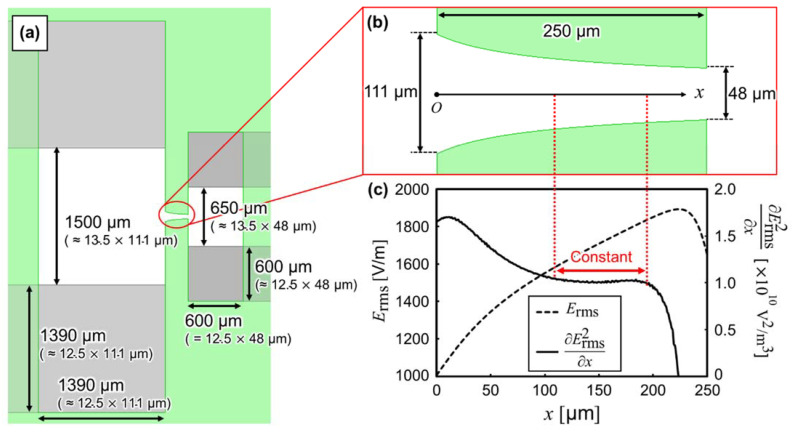
(**a**) Top view of the designed electrode shape of isomotive iDEP creek-gap device. (**b**) Top view of the designed shape of the creek-gap insulators. (**c**) Distribution of the magnitude of electric field, $E_{\mathrm{rms}}$, and $\partial E_{\mathrm{rms}}^{2}$ /∂x on the centerline of the creek-gap insulators. $\partial E_{\mathrm{rms}}^{2}$ /∂x was approximately constant within ± 2.5%, in the range of *x* = 110 µm to 195 µm.

Figure 4a,b show the design guideline of electrodes and creek-gap insulators, respectively. The electrodes and insulators were designed as mentioned above. The creek-gap insulators were calculated using Equation (10). Figure 4c shows the distribution of numerically calculated $E_{\mathrm{rms}}$ and $\partial E_{\mathrm{rms}}^{2}$/∂x when the electrodes apply a voltage of 1 $V_{\mathrm{rms}}$. Furthermore, $\partial E_{\mathrm{rms}}^{2}$/∂x was approximately constant within ±2.5%, in the range of *x* = 110 µm to 195 µm. In contrast, it was approximately constant within ±25% in the previous study [23]. In the experiment, the silicone rubber cover was changed to dimethylpolysiloxane, due to the promotion of the adhesion between the insulator and cover.

## 3. Materials and Methods

### 3.1. Fabrication of the Isomotive iDEP Device

#### 3.1.1. Patterning of the Electrodes with Conventional Process

We used a glass wafer of 2 inch-diameter and 0.5 mm-thickness as the device’s substrate. The aluminum of 400 nm-thickness was deposited using vacuum vapor deposition (SVC-700TMSG, Sanyu Electron, Tokyo, Japan) and coated with the positive photoresist (S1805G, The Dow Chemical Company, Midland, MI, USA). After pre-bake on a hot plate at 90 °C for 3 min, the photoresist was exposed to the electrode pattern using Maskless Aligner (MLA150, Heidelberg Instruments, Heidelberg, Germany). The exposed photoresist was developed using 2.2% tetramethylammonium hydroxide (TMAH; MF-319, The Dow Chemical Company, Midland, MI, USA) and post-baked at 130 °C for 3 min. The aluminum electrode, uncovered with photoresist, was etched by mixed acid (Kanto Chemical, Tokyo, Japan) at 40 °C. Then, the photoresist was removed using AZ REMOVER 100 (Merck, Darmstadt, Germany).

#### 3.1.2. Patterning of the Insulators with Backside Exposure

**Figure 5:**
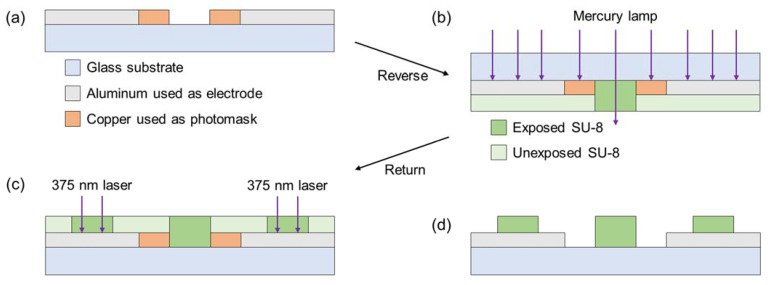
Illustrations of the patterning of insulators with backside exposure. (**a**) Patterning of the copper used as a photomask. (**b**) Backside exposure using the mask aligner. SU-8 covered with aluminum or copper is unexposed. (**c**) Selective exposure using the Maskless Aligner. (**d**) After developing SU-8, the copper photomask was etched.

The fabrication of the insulators should be highly accurate and consistent with the designing guideline (shown in Figure 4), in order to obtain a constant $\partial E_{\mathrm{rms}}^{2}$/∂x. The insulator was fabricated using backside exposure to form close to the design guideline (Figure 5).

The copper of 250 nm-thickness, used as photomask, was deposited on the substrate; these processes were the same as aluminum electrodes (Figure 5a). The copper was etched using Pure Etch C200 (Hayashi Pure Chemical, Osaka, Japan). After copper patterning, the substrate was baked as dehydration at 200 °C for 5 min. The baked substrate was coated with hexamethyldisilazane (HMDS; OAP, Tokyo Ohka Kogyo, Kanagawa, Japan) to promote adhesion between the glass substrate and insulator. Then, it was baked at 200 °C for 5 min. The negative photoresist (SU-8 3050, Kayaku Advanced Materials, Westborough, MI, USA), used as an insulator, was dripped 2 mL and coated by a spin coater to obtain an insulator with a thickness of 70 µm. The photoresist was soft-baked at 65 °C and 95 °C for 5 and 45 min, respectively. Then, it was cooled on the hot plate to room temperature (23 °C). The exposure was conducted in two steps, as follows: (1) the exposure of the photoresist on the glass from the backside of the substrate using Mask Aligner (BA100, Nanometric Technology, Tokyo, Japan) (Figure 5b); (2) exposure of the photoresist on the aluminum electrode from the topside of the substrate using Maskless Aligner (Figure 5c). The photoresist was post-baked at 65 °C and 95 °C for 5 min each; it was cooled on the hot plate to room temperature. In the photoresist development, we used propylene glycol methyl ether acetate (PGMEA)-based developer (SU-8 Developer, Kayaku Advanced Materials, Westborough, MI, USA). The developed photoresist was hard-baked at 65 °C for 5 min, 95 °C for 5 min, and 130 °C for 30 min; it was cooled on the hot plate to room temperature. The copper photomask was etched using the Pure Etch C200 (Figure 5d).

**Figure 6:**
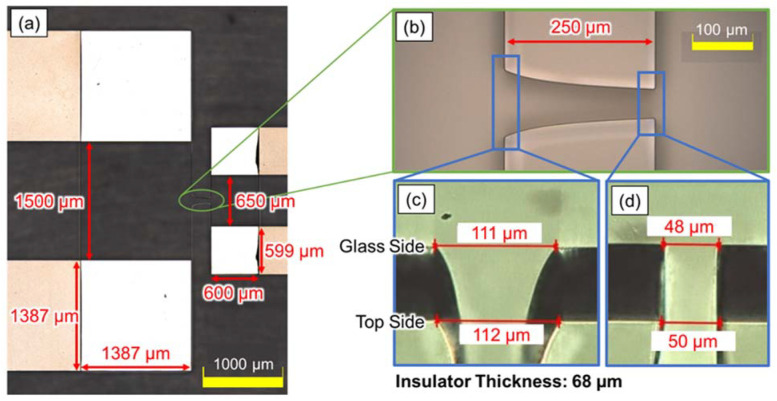
Microscope image of the fabricated device. The shape of the fabricated device was mostly consistent with the designed parameters shown in Figure 4. (**a**) The entire chamber of the device, with the objective lens focused on the electrode. (**b**) The enlarged image of the creek-gap insulator, with the objective lens focused on the top of the insulator. (**c**) The depth synthetic image of the narrower insulator gap. (**d**) The depth synthetic image of the wider insulator gap.

Figure 6 shows the image of the fabricated device. Figure 6a,b were captured using a microscope (OLS4500, Olympus, Tokyo, Japan), and Figure 6c,d show the depth synthetic image, captured by tilting the device with a high-speed microscope system (VW-9000, Keyence, Osaka, Japan). The shape of the fabricated device was mostly consistent with the designed parameters, shown in Figure 4.

### 3.2. Measurement of the Particle Velocity and Device Impedance

We used monosized polystyrene particles of 20 µm-diameter (4220A, Thermo Fisher Scientific, Waltham, MA, USA) to measure the particle velocity, due to their uniformity and homogeneity. The diameter of the particles had the coefficient of variation of 1.1 % (written on the package). The particles were suspended in deionized (DI) water, with the conductivity of 0.3 mS/m, then filled in the chamber. The conductivity of DI water was measured using a conductivity meter (DS-72E, HORIBA, Ltd., Kyoto, Japan). A voltage was applied to the electrodes using a function generator (WF1968, NF Corp, Kanagawa, Japan), and the applied voltage was measured using an oscilloscope (GDS-2104, GW Instek, New Taipei City, Taiwan). The particle behavior and position were observed and recorded using a high-speed microscope system (VW-9000, Keyence, Osaka, Japan), with the frame rate of 250 fps. The particle velocities were measured using motion analyzer software with the microscope system.

Additionally, we used an impedance analyzer (4294A, Agilent Technologies, Santa Clara, CA, USA) to measure the device impedance. Prior to the measurement, the impedance analyzer was short- and open-calibrated by short- and open-circuited wires, respectively. The impedance was measured at 201 points on a logarithmic scale, from 40 Hz to 100 MHz, at a signal voltage of 0.5 V_rms_. The measurement results were recorded to the floppy disk and analyzed on a personal computer. The conductivity of the medium was adjusted by mixing phosphate-buffered saline (PBS; 314-90185, Nippon Gene, Tokyo, Japan).

## 4. Results and Discussion

### 4.1. Distribution of the Particle Velocity

The electrical properties of particles or biological cells are evaluated by $\partial E_{\mathrm{rms}}^{2}$/∂x. $\partial E_{\mathrm{rms}}^{2}$/∂x is numerically calculated from the electromagnetic field simulation software. Therefore, the precision of the calculation is directly affected, in order to evaluate the electrical properties. In this section, we confirmed the calculation precision by comparing the calculated $\partial E_{\mathrm{rms}}^{2}$/∂x and measured particle velocity.

**Figure 7:**
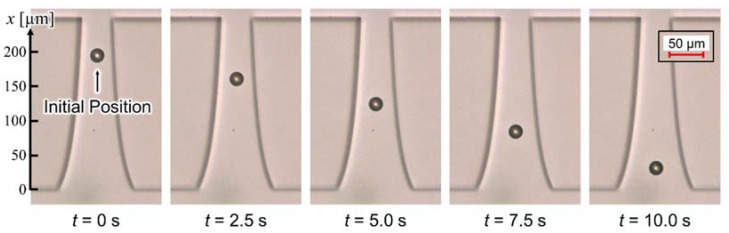
Behavior of the nDEP-exhibited particle. The applied voltage and frequency were 40 V_p-p_ and 1 MHz, respectively. The particle individually moved on the centerline between insulators without levitation.

**Figure 8:**
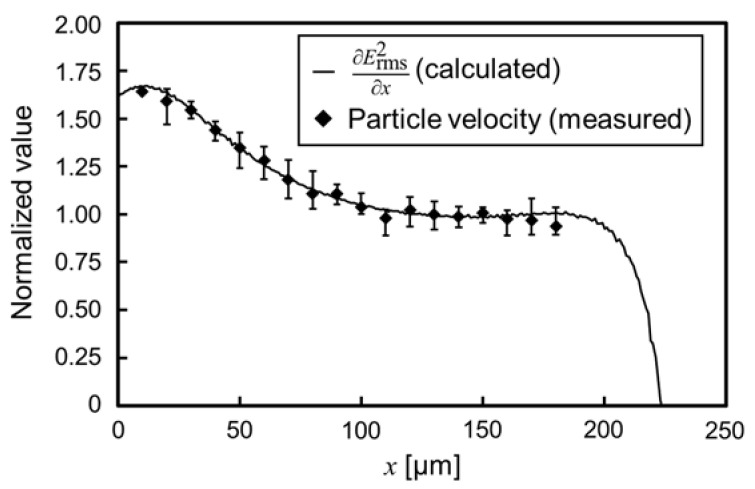
Distribution of the calculated $\partial E_{\mathrm{rms}}^{2}\;$/∂x and measured particle velocity. The applied voltage and frequency were 40 V_p-p_ and 1 MHz, respectively. The error bar of the plot shows the maximum and minimum velocity in the measurement.

Figure 7 shows the behavior of the nDEP-exhibited particle. The applied voltage and frequency were 40 V_p-p_ and 1 MHz, respectively. The particle moved on the centerline between insulators at the single-particle level. Before applying the voltage, the particle settled down on the substrate, and the particle was driven by the DEP force, without levitation, after applying the voltage. Figure 8 shows the distribution of $\partial E_{\mathrm{rms}}^{2}$/∂x and particle velocity. One particle was measured at a time, and five particles were, thus, measured. The error bar of the plot shows the maximum and minimum velocity in this measurement. Both values were normalized at the position of 130 µm for comparison, since the particle velocity is proportional to $\partial E_{\mathrm{rms}}^{2}$/∂x, according to Equation (9). At the position of 130 µm, $\partial E_{\mathrm{rms}}^{2}$/∂x was 2.04 × 10^12^ V^2^/m^3^, and the particle velocity was 19.3 µm/s. The distribution of $\partial E_{\mathrm{rms}}^{2}$/∂x was consistent with that of the particle velocity. This result indicates that $\partial E_{\mathrm{rms}}^{2}$/∂x was calculated with high precision, and it is possible to evaluate the electrical properties of particles using the calculated $\partial E_{\mathrm{rms}}^{2}$/∂x. Equation (9) assumes that the particle moves on a constant $\partial E_{\mathrm{rms}}^{2}$/∂x. However, the time constant shown in Equation (8) is sufficiently small, which it is considered negligible. Moreover, the distribution of particle velocity from 110 to 180 µm was approximately constant. This velocity profile means that the particles moved on the isomotive field. This isomotive movement enables particle characterization with straightforward measurement.

### 4.2. Particle Velocity against Applied Frequency

In this section, we discussed the measurement result of the frequency property of particle velocity. We applied a sinusoidal voltage of 40 V_p-p_ and frequency from 10 Hz to 20 MHz to the electrode. Additionally, we measured the velocity of five particles at each frequency. Then, the velocity was calculated from the travel time, using *x* = 135 to 115 µm, as shown in Figure 4 and Figure 7. At frequencies of 10, 30, and 60 Hz, the particle tracking software of the microscope system was used to measure the particle velocity because the particles moved, vibrating in time with the sinusoidal wave, and the average velocity was calculated from the tracked velocity. In all frequencies, the particle exhibited nDEP because the permittivity and conductivity of polystyrene are lower than DI water.

**Figure 9:**
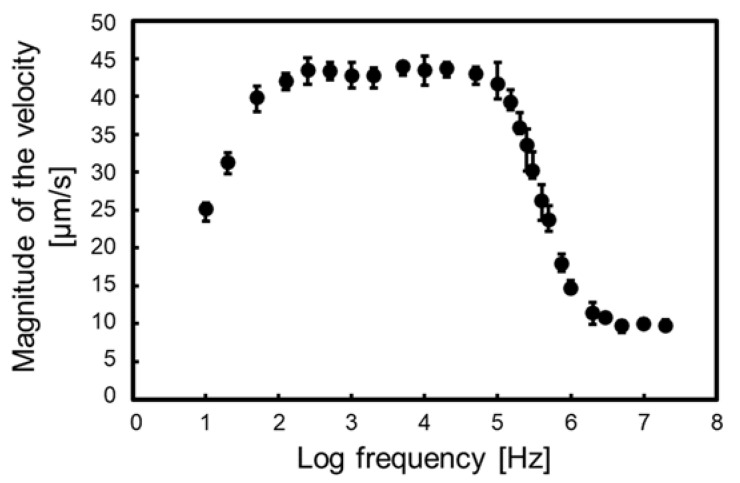
Particle velocity against the applied frequency. The velocity was measured from 10 Hz to 20 MHz. The error bar of the plot shows the maximum and minimum velocity in the measurement. The velocity was calculated from the travel time, using *x* = 135 to 115 µm, as shown in Figure 4 and Figure 7.

Figure 9 shows the particle velocity against the applied frequency. The error bar of the plot shows the maximum and minimum velocity in the measurement. The velocity measurement of a wide frequency band was performed using the proposed isomotive iDEP device. Generally, the velocity measurement using eDEP devices is not easy below 10 kHz because of the EDL between the medium and electrode. However, the influence of the EDL in iDEP occurs at a very low frequency because the electrode distance of the iDEP device is longer than that of the eDEP device. In the proposed device, the influence occurs below 100 Hz. Further discussions are described in the next section.

According to Equation (9), the electrical properties of the particles are reflected in the frequency property of the particle velocity by Re[CM*]. The Re[CM*] of the homogeneous particles has a low- and high-frequency limit and is dominated by conductivity and permittivity, respectively [28]. The limit values were at 100 Hz to 100 kHz and 5 MHz to 20 MHz.

The DEP-induced particle velocity is dependent on the unique electrical properties of the particles, such as biological cells, and can be utilized to identify the cell types. Additionally, the new DEP properties of biological cells at low frequencies, through single-cell analysis, can be revealed by measuring the cell velocity using the proposed device.

### 4.3. Impedance Measurement of the Proposed Device

**Figure 10:**
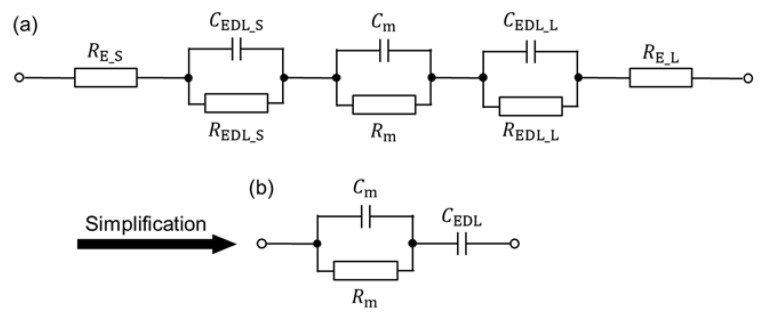
Equivalent circuit of the proposed device. (**a**) Equivalent circuit, considering elements consisting of the device at the measurement frequency. (**b**) Simplified equivalent circuit.

In this section, the impedance of the proposed device was investigated to elucidate the influence of the EDL at the interface between the medium and electrode at low frequency. Figure 10a shows the equivalent circuit, considering elements consisting of the device at the measurement frequency. Here, *R*_E_, *R*_EDL_, and *R*_m_ are the resistance of the electrode, EDL, and medium, respectively; *C*_EDL_ and *C*_m_ are the capacitance of the EDL and medium, respectively. The subscript of “__S_” and “__L_” mean the electrodes with a smaller and larger area, respectively, at the following conditions:

- *R*_E_S_ and *R*_E_L_ are negligible: *R*_E_S_ and *R*_E_L_ are much smaller than *R*_m_;
- *R*_EDL_S_ and *R*_EDL_L_ are open-circuited: assuming that there is no charge movement in the EDL.

The equivalent circuit is simplified as shown Figure 10b.

**Figure 11:**
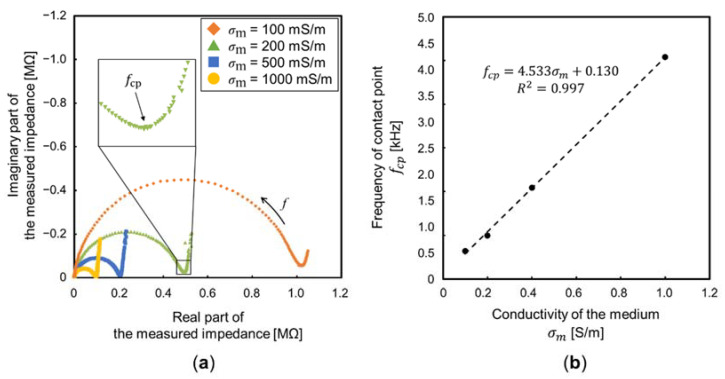
Measurement results of the proposed device. (**a**) Complex impedance locus with different $\sigma_{m}$. The diameter of the semi-circle is proportional to the ${\sigma_{m}}^{-1}$. (**b**) The frequency, which is the contact point between the semi-circle by the medium and locus by the EDL against $\sigma_{m}$.

The impedance measurement using the medium of low conductivities was impossible due to the limitation of the impedance analyzer. Accordingly, the influence of the EDL, when DI water was used, was predicted by the impedance measurement results, using the medium of high conductivities. Figure 10a shows the measurement results, expressed by a complex impedance locus (also called Cole–Cole plots). At a very high frequency, the measured impedance was nearly 0 Ω because *C*_m_ and *C*_EDL_ were considered short-circuited. As the frequency decreased, the impedance of the medium was observed, due to *C*_m_≪*C*_EDL_. The locus of a parallel circuit, consisting of a resistance and capacitance, is represented by a semi-circle. With further decrease in the frequency, the component of the imaginary part was significantly decreased, and the measured impedance was approximately equal to *R*_m_. The diameter of the semi-circle is proportional to ${\sigma_{m}}^{-1}$ (Figure 11a). At a very low frequency, the impedance of *C*_EDL_ was measured with the sum of *R*_m_. Accordingly, the EDL influences the measurement of particle velocities below the frequency, which is the contact point between the semi-circle by the medium and locus by the EDL. Figure 11b shows the frequencies of the contact point, *f*_cp_, against the conductivity of the medium. There was a linear relationship between *f*_cp_ and $\sigma_{m}$. Additionally, *f*_cp_ (predicted by the linear approximation) is 131 Hz at the conductivity of DI water (0.3 mS/m). This frequency was almost consistent with the frequency where particle velocity began to decay; thus, suggesting the decay is due to the EDL. At the frequency below *f*_cp_, the equivalent circuit of the proposed device is more simplified as a series circuit of *R*_m_ and *C*_EDL_. The cut-off frequency, *f*_co_, where the square of the voltage of *R*_m_ is half of the square of the voltage applied to the device, is given by:

(11) $${\;f}_{\mathrm{co}}=\frac{1}{{2\pi C}_{\mathrm{EDL}}R_{m}}$$

According to Equation (11), *f*_co_ decreases as $R_{m}$ increases. In the iDEP device, $R_{m}$ is higher than the general eDEP devices because of the distance between electrodes, which is applied via high and low voltage in the iDEP device, is longer than that of the eDEP devices. Therefore, the particle velocities at a wide frequency band, including low frequency, were measured in the iDEP device.

## 5. Conclusions

In this paper, we proposed the isomotive iDEP creek-gap device for single-cell analysis. The design guideline of the proposed device was based on the observations from the generated electric field properties of the simplified iDEP device. The $\nabla E_{\mathrm{rms}}^{2}$ generated on the centerline between the creek-gap insulators is more constant than that of the previous device. Furthermore, the distribution of the measured particle velocity was consistent with that of the calculated $\nabla E_{\mathrm{rms}}^{2}$. Additionally, the wide frequency band measurement of the particle velocity was performed from 10 Hz to 20 MHz. The velocity is decayed below 100 Hz, due to the EDL between the electrode and medium. This is explained using the equivalent circuit model of the proposed device, with the impedance measurement. These results suggest that various particle or cell properties are obtained by simple measurement using the proposed device.

Because of the simple principle, inexpensive fabrication, and high versatility of isoDEP, it is considered a major method of single-cell analysis and micromaterial evaluation. However, the proposed device has challenges with low throughput. Thus, new explorations are urgently needed that combine microfluidics, multiple arrays, automatic video processing, and frequency sweeping.

## Data Availability

Not applicable.
